# Beta diversity patterns reveal positive effects of farmland abandonment on moth communities

**DOI:** 10.1038/s41598-018-38200-3

**Published:** 2019-02-07

**Authors:** Murilo Dantas de Miranda, Henrique M. Pereira, Martin F. V. Corley, Thomas Merckx

**Affiliations:** 1 Institute of Biology, Martin Luther University Halle Wittenberg, Halle (Saale), Germany; 2 German Centre for Integrative Biodiversity Research (iDiv) Halle-Jena-Leipzig, Leipzig, Germany; 3 Infraestruturas de Portugal Biodiversity Chair, CIBIO/InBIO - Research Network in Biodiversity and Genetic Resources, Instituto Superior de Agronomia, Universidade de Lisboa, Tapada da Ajuda, Lisboa, Portugal; 4 CIBIO - Research Center in Biodiversity and Genetic Resources, Universidade do Porto, Campus Agrário de Vairão, Vairão, Portugal; 5 Behavioural Ecology and Conservation Group, Biodiversity Research Centre, Earth and Life Institute, Université catholique de Louvain (UCLouvain), Louvain-la-Neuve, Belgium

## Abstract

Farmland abandonment and the accompanying natural succession are largely perceived as unwanted amongst many European conservationists due to alleged negative effects on biodiversity levels. Here, we test this assumption by analysing alpha, beta and gamma diversity patterns of macro-moth communities in habitats on an ecological succession gradient, from extensively managed meadows to scrub-encroached and wooded sites. Macro-moths were light-trapped at 84 fixed circular sampling sites arranged in a semi-nested design within the National Park of Peneda-Gerês, NW-Portugal. In total, we sampled 22825 individuals belonging to 378 species. Alpha, beta and gamma diversity patterns suggest that farmland abandonment is likely to positively affect both overall macro-moth diversity and forest macro-moth diversity, and to negatively affect species diversity of non-forest macro-moth species. Our results also show that spatial habitat heterogeneity is important to maintain gamma diversity of macro-moths, especially for rare non-forest species and habitat specialists.

## Introduction

Land-use change has been pinpointed as one of the main factors of global biodiversity change^1^, reducing species diversity at various spatial scales and modifying species interactions within ecological communities^2,3^. However, land-use change is not always negative for biodiversity, as it can also include ecological restoration from intense human land-use back to a more natural state^4^.

Here, we focus on farmland abandonment, which is a type of land-use change whose effects on biodiversity are currently unresolved. Whilst abandonment is perceived as a threat to biodiversity amongst many European conservation biologists^5–7^, some see the accompanying ecological succession as an opportunity for ecological restoration with positive effects on biodiversity^8,9^. We here test which of both assumptions is correct by analysing diversity patterns of macro-moth communities in three habitat types on a gradient of ecological succession, representing a space-for-time chronosequence of farmland abandonment.

The focus of our study is on macro-moths –an abundant and species-rich group of insects– as they react swiftly to environmental change and play key roles in ecosystem functioning, for example in terms of prey resource, nutrient cycling and nocturnal pollination^10^. Beta diversity –the spatial change in species composition– is considered a key concept to understand how local community assembly (alpha diversity) is linked to the regional species pool (gamma diversity)^11,12^. Because a comprehensive analysis of these three aspects of diversity is needed to fully understand the biodiversity consequences of farmland abandonment, we thus compare alpha, beta and gamma diversity levels of macro-moths amongst three habitat types: extensively managed meadows and both scrub-encroached sites and native woodland. We also separate analyses contrasting forest versus non-forest species, as both groups are expected to show markedly different responses to farmland abandonment. Because abandonment is associated with more advanced stages of ecological succession, our hypothesis is that farmland abandonment positively affects overall moth diversity, but may negatively affect species diversity of early-successional species groups. Because an increased variety in habitat types (i.e. habitat heterogeneity), providing for several successional stages, is known to benefit gamma diversity at the landscape-scale of moths overall^13^, we hypothesise that the provision of early-successional biotopes within forest-dominated landscapes is essential both to counter these negative effects for early-successional species groups and to reach high gamma diversity levels at the landscape scale.

## Results

### Gamma diversity

**Figure 1:**
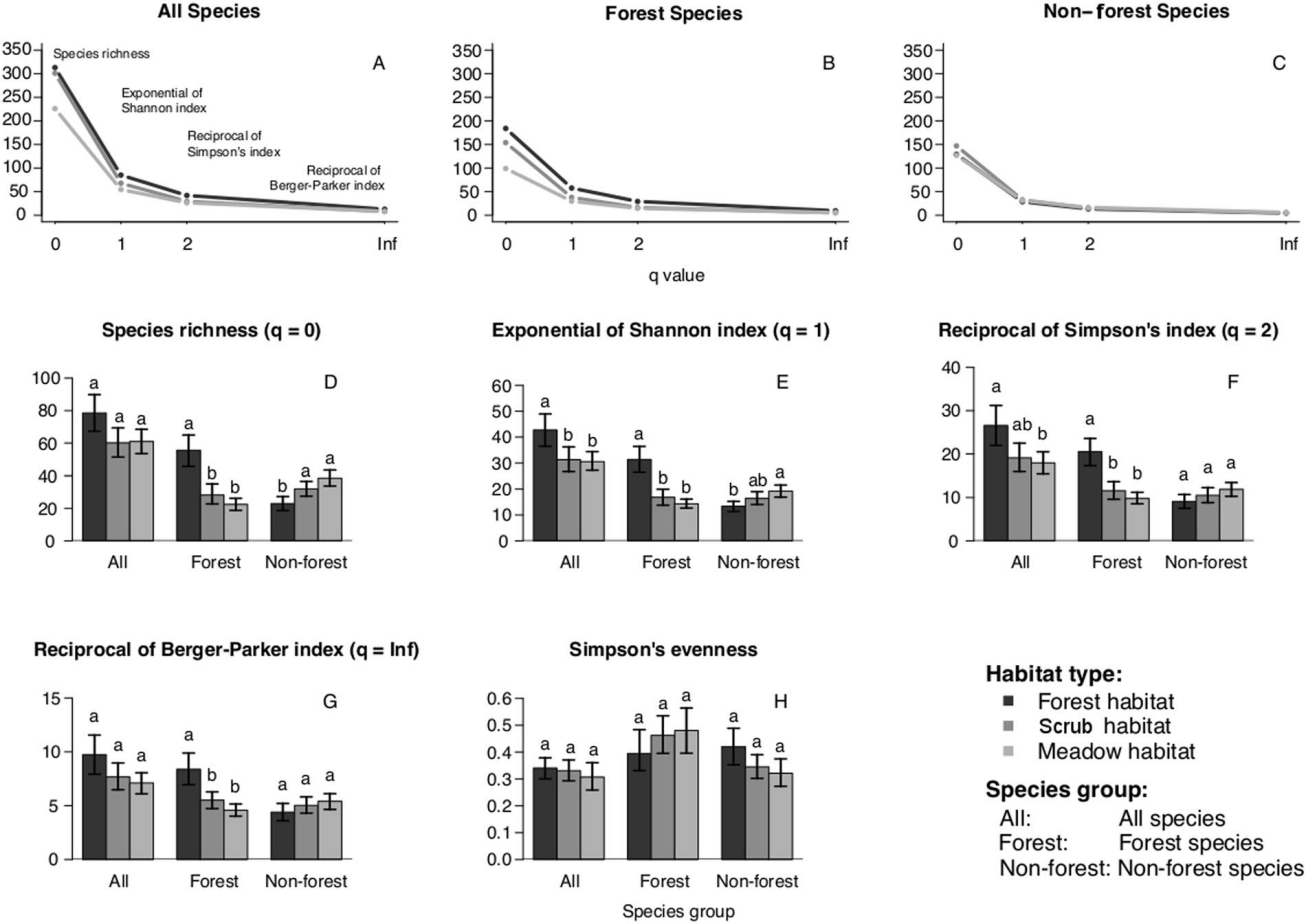
Comparison of effective species numbers between habitats. Effective species numbers are based on the same generalized entropy formula, differing only by an exponent *q* which varies between 0 and positive infinity. Low values indicate that rare species are taken into account, while high values indicate that abundant species are taken into account: species richness (*q* = 0), the exponential of Shannon diversity (*q* = 1), the reciprocal of Simpson’s diversity (*q* = 2), the reciprocal of the Berger-Parker index (*q* → $\infty$). (**A**) Effective species numbers at regional scale for all macro-moth species, (**B**) for forest species and (**C**) for non-forest species. (**D**) Mean species richness, (**E**) mean exponential of Shannon index, (**F**) mean reciprocal of Simpson’s index, (**G**) mean reciprocal of Berger-Parker index and (**H**) mean Simpson’s evenness for the three species groups at the local scale. Dark-, medium- and light-grey colours represent forest, scrub and meadow habitats, respectively. Error bars are 95% confidence intervals based on 4000 bootstrap replicates. Different letters represent statistically significant differences.

A total of 22825 macro-moth individuals from 378 species were sampled (see Supplementary Table S1). Gamma diversity was highest in forest (313 species, 8355 individuals), intermediate in scrub (301 species, 9870 individuals), and lowest in meadow habitat (226 species, 4600 individuals). The gamma diversity profiles (effective species numbers) showed highest overall and forest macro-moth diversity values at forest sites, intermediate values at scrub sites, and lowest values at meadow sites. In contrast, effective species numbers of non-forest macro-moths appeared less affected than those for forest moths and moths overall, at least when contrasting scrub versus meadow habitat (Fig. 1A–C). We note that while the number of meadow sampling sites (n = 17) was the smallest, the number of scrub sites was highest (n = 41), with an intermediate value for forest sites (n = 26). Thus, gamma diversity of scrub may have been slightly overestimated when compared with the meadow and forest habitats, although the difference in gamma diversity of forest in relation to the meadow habitat is too large to be explained by our heterogeneous sampling effort alone. The latter is also clear from the higher accumulated species richness for forest compared to meadow habitat (Supplementary Fig. S1).

### Alpha diversity

Overall, there was no difference in species richness nor in the Berger-Parker index’s reciprocal among the three habitat types (Fig. 1D,G). However, both the Shannon index’s exponential and the Simpson’s index’s reciprocal were higher in forest than in meadow habitat, with scrub habitat values similar to meadow habitat values for the Shannon index’s exponential and not dissimilar from both forest and meadow habitat values for the Simpson’s index’s reciprocal (Fig. 1E,F).

For forest species, forest habitat was characterised by higher species richness and higher Shannon index’s exponential, Simpson’s index’s reciprocal and Berger-Parker index’s reciprocal than both scrub and meadow habitat, which did not differ from each other (Fig. 1D–G).

For non-forest species, species richness and Shannon index’s exponential were higher in meadow than in forest habitat, with scrub habitat values similar to meadow habitat values for species richness and not dissimilar from both meadow and forest habitat values for Shannon index’s exponential (Fig. 1D,E). There was no difference in Simpson’s index’s reciprocal nor in Berger-Parker index’s reciprocal among the three habitat types (Fig. 1F,G).

Simpson’s evenness did not differ among the three habitat types, neither overall, nor for forest and non-forest species separately (Fig. 1H).

### Beta diversity: within-habitat variation in species composition

**Figure 2:**
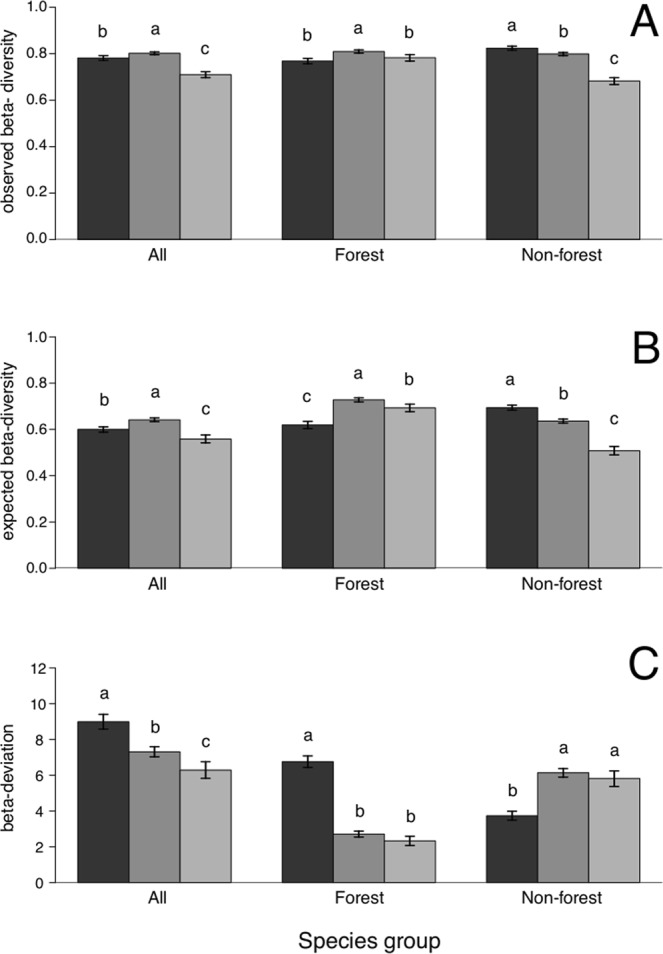
Beta diversity across habitats and species groups. (**A**) Observed beta diversity (*in casu* Jaccard index), (**B**) expected beta diversity from a null model based on random sampling from the regional species pool, and (**C**) beta deviations (standardized effect sizes which represent the difference between observed beta diversity and expected beta diversity) for all macro-moth species (left), forest species (centre), and non-forest species (right). Beta deviations are positive, indicating beta diversity higher than expected by chance. Dark-, medium- and light-grey bars represent forest, scrub and meadow habitats, respectively. Error bars are 95% confidence intervals based on 4000 bootstrap replicates. Different letters represent statistically significant differences. Similar results were obtained using the Sørensen index (see Supplementary Fig. S2).

Within-habitat beta diversity, observed for macro-moths overall, was slightly higher in scrub than in forest, and was lowest in meadow habitats (Fig. 2A). This pattern is similar to the expected beta diversity pattern. Nevertheless, the observed beta diversity was consistently higher than the expected beta diversity. This suggests a higher dissimilarity between communities from different locations of the same habitat than what would be expected by chance based upon the observed alpha and gamma diversity of these habitats (Fig. 2A,B). The positive beta deviation values for all three habitat types do indeed show that their beta diversity was much higher than what would be expected from a random sampling model, but now with the highest value for forest, an intermediate value for scrub, and the lowest value for meadow habitat (Fig. 2C). This means that the compositional variation of macro-moths overall was largest in forest and smallest in meadow habitats, after accounting for the observed alpha and gamma diversity. The latter was also confirmed by the habitat-specific species accumulation curves, which were steeper for forest than for meadow habitat (Supplementary Fig. S1).

Although forest species had lowest observed and expected beta diversity in forest habitat (Fig. 2A,B), their beta deviation was almost three times higher in forest than in scrub and meadow habitat (Fig. 2C). Similarly, non-forest species displayed lowest observed and expected beta diversity in meadow habitat and intermediate values in scrub habitat (Fig. 2A,B), whilst their beta deviations at meadow and scrub habitat were almost double the value at forest habitat (Fig. 2C). Hence, compositional variation –corrected for alpha diversity– was highest in forest habitat for forest species, whilst it was highest at both scrub and meadow habitat for non-forest species.

### Beta diversity: spatial turnover rates

**Figure 3:**
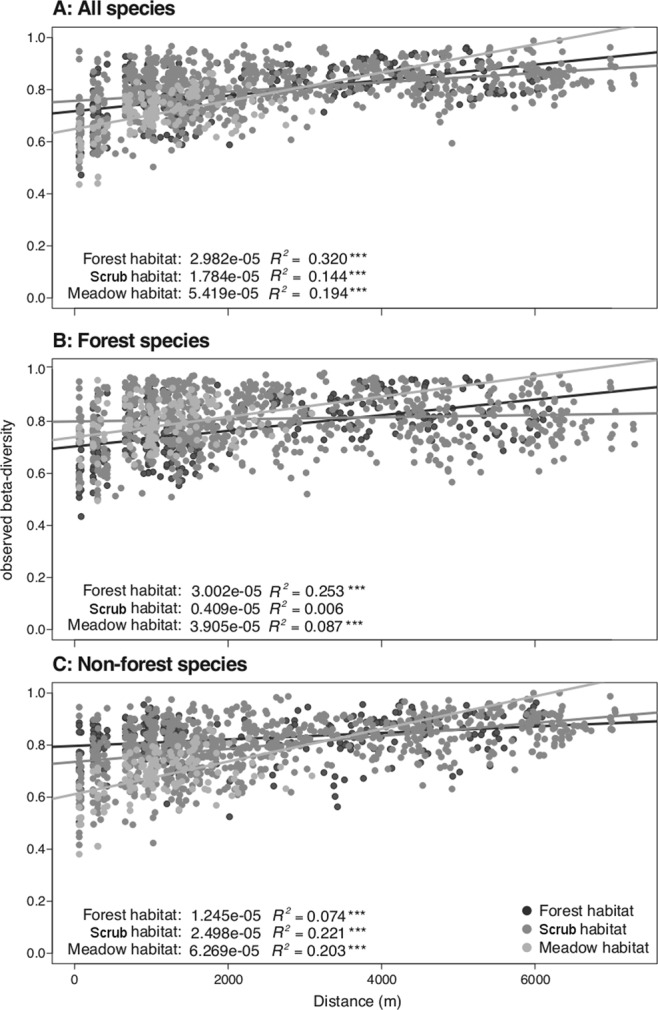
Relationships between geographic distance and beta diversity (*in casu* Jaccard index) for pairs of sampling sites across habitats and species groups. (**A**) Observed beta diversity for all macro-moth species, (**B**) forest species, and (**C**) non-forest species. Dark-, medium- and light-grey dots represent forest, scrub and meadow habitats, respectively. The solid lines represent the best-fit lines from linear regression. The turnover rates (slopes), *R*^*2*^-values and significance levels of turnover rates (***P < 0.001, **P < 0.01 and *P < 0.05) are given within each panel. Similar results were obtained using the Sørensen index (see Supplementary Fig. S3).

Overall, observed beta diversity of macro-moth communities increased with increasing distance between sites. This positive turn-over rate pattern –for all species lumped– was strongest for meadow, intermediate for forest, and weakest for scrub habitat (Fig. 3A). Forest species displayed high turn-over in both forest and meadow habitat, whilst no turn-over in scrub habitat (Fig. 3B). Turn-over of non-forest species was highest for meadow, intermediate for scrub and lowest for forest habitat (Fig. 3C).

## Discussion

Having compared species diversity of macro-moth communities in three different habitats, representing a space-for-time chronosequence gradient of farmland abandonment, we found –using a range of indices– that both alpha and beta diversity were overall considerably higher in forest than in both scrub and meadow habitats. Nevertheless, meadow habitat showed the highest spatial turnover rate, although forest habitat was characterised by a high turnover rate too, especially so for forest species. Because our aim is broad-scale, focusing on alpha, beta and gamma diversity patterns at once, we address elsewhere the quantification of the effect of habitat amount in the directly surrounding landscape on local diversity (Merckx, Dantas de Miranda & Pereira, *under review*). So, although we admit that some of the variation in local diversity was due to spillover^14^ effects and adjacent biotope context, we believe that the number of sampled sites for each habitat type was sufficiently large to allow a relevant, real-landscape representation of the existing variation in local diversity for each habitat type, and this within each of the landscape types differing in relative cover of the three habitat types.

The alpha diversity profiles indicate that farmland abandonment is likely to positively affect the gamma diversity of macro-moths overall. These findings strongly suggest that multi-habitat landscapes, characterised by a substantial heterogeneity of meadow, scrub and forest habitat, are able to reach high levels of gamma diversity for macro-moths, also because many species are able to use several habitat types to a varying extent. In the same vein, Merckx *et al*.^13^ showed that the retention of open habitats within English broadleaved woodlands increases the gamma diversity of macro-moths, since they allow open-biotope specialists to occur, even though their alpha diversity is much lower than the alpha diversity of the closed woodland habitat specialists. Similarly, and especially given the high spatial turnover rate for meadows, this habitat too is important for gamma diversity levels at the landscape and regional scales. A Finnish study comparing butterfly and day-active moth communities in continuously-grazed versus abandoned meadows came to an identical conclusion, since on the one hand species richness and total abundance were highest in abandoned meadows, both overall and specifically for meadow species, while on the other hand diversity and evenness indices were in most cases highest in continuously-grazed meadows^15^. As such, these and our findings show the importance of retaining habitat diversity during farmland abandonment. Whilst abandonment poses risks regarding the long-term presence of meadow habitats, especially if the process happens all at once within the whole landscape, grazing is able to cater for sufficient landscape heterogeneity including meadows. This can be achieved via restorative grazing by cattle or via rewilding with large grazers^15–17^.

At local and landscape scales, forest habitat displayed higher alpha and gamma diversity than both scrub and meadow habitat. Similarly, Beck *et al*.^18^ showed that primary and old-grown regenerated forest sites in Borneo are characterised by a much higher diversity of geometrid moths than agricultural sites. Kivinen *et al*.^19^ too found that forest cover correlated positively with macro-moth species richness, whilst agricultural field cover correlated negatively, demonstrating that overall species richness was lower in homogenous agricultural landscapes. However, when the focus is on non-forest species, we show that their alpha diversity and compositional variation among sites, as expected, decreases with ecological succession; the highest diversity was observed in meadow habitat, with lower but statistically similar levels for scrub, and lowest diversity in forest habitat. By contrast, intensification of meadow habitats and agricultural landscapes is known to drastically reduce overall moth diversity levels^20^. One aspect of agricultural intensification is a reduction in woody cover, which is known to be of importance at the landscape scale for moths, even for open-biotope species^19,21,22^. Trees and shrubs indeed provide essential resources to open-biotope species too, such as a more sheltered micro-climate^23–25^.

Our results show that the inter-habitat differentiation in species diversity –both for all species and forest species only– occurred for all *q* values, which indicates that the differentiation occurred for both rare and common species. For non-forest species however, the inter-habitat differentiation only occurred at low values, indicating that the diversity differences are due to differences in the diversity of rare species only. Summerville *et al*.^26^, who also used the first three *q* values in order to study spatial variation in species diversity and composition of North American forest Lepidoptera, found that species richness changed equally across all spatial scales due to the large amount (>50%) of rare species.

Although the observed beta diversity for all species was higher in scrub than in forest and meadow habitats, the pattern completely changed after taking into account gamma and alpha diversity, with the highest beta deviation for forest, intermediate beta deviation for scrub, and the lowest beta deviation for meadow habitat. Hence, the forest habitat is not only characterised by the highest gamma and alpha diversity, but by the highest beta diversity too. An explanation for this high forest beta diversity is that although the native forest habitat in our study area is largely composed of *Quercus robur* and *Q*. *pyrenaica*, many other tree and plant species are part of the native forest community. The spatial heterogeneity and vertical layering of host-plant species within forest habitat can lead to a high variation in community composition between sampling sites^27^. Nonetheless, the meadow habitat displayed the steepest spatial turnover rate overall, but it was largely explained by the distribution of rare non-forest species, which although important for conservation in their own right may be less important in terms of their contribution to natural processes and ecosystem functioning^28,29^.

Our results also showed that the observed beta diversity was consistently larger than the beta diversity as expected by chance, which translated in positive beta deviation values for all habitat types and species groups. This observation suggests that the size of the regional species pool (i.e. gamma diversity) for the three habitat types cannot entirely explain the geographical distribution of their beta diversity. Thus, this suggests that macro-moth communities are additionally shaped by ecological assembly mechanisms (e.g., habitat filtering and biological interactions) and/or random processes (e.g., dispersal, colonization and extinction), producing higher beta diversity^30,31^.

We measured beta diversity using two approaches, each one with its own limitations. While in the first approach we calculated observed beta diversity and related it to distance between sites, the second approach used a null model that allowed us to examine the effect of turnover while controlling for each site’s species richness, which is the result of local processes. Although the first approach fully accounts for spatial clustering of sites in the calculation of turnover, the linear regression assumptions of independence are violated^2^. However, this violation is mainly important when comparing significance tests of different regression lines, and particularly so for results that are borderline significant. Here, the significance levels are anyway so high that they are likely robust to this violation of independence, while the significance tests are not the main message of our analysis. Instead, our main message is that the spatial turnover of species composition was highest for the meadow habitat. Complementary to this, the second approach allows estimating the strength of the beta diversity between sites, and although this approach does not account for spatial clustering of sites, results are likely to be robust to such clustering. It shows that in all habitats beta diversity is higher than what is expected by a random null model. Particularly, it suggests that forest is key for beta diversity of forest species, whereas scrub and meadows seem to have more similar roles regarding the beta diversity of non-forest species.

It is important to understand how farmland abandonment affects native biodiversity to mitigate its negative ecological impacts. Although we here demonstrate that a late stage of ecological succession following farmland abandonment (i.e. native woodland) is characterised by the highest diversity levels of macro-moths, our results and the results of previous studies also show the importance of retaining sufficient spatial variety in habitat types for macro-moth diversity, especially for rare non-forest species^13,15,32^. Mitigation may entail the release of large grazers when this habitat heterogeneity in abandoned regions has become too low, with open, early-successional land cover types almost absent. Such low heterogeneity may occur if natural disturbances, such as grazing, happen to be insufficient early on in the passive rewilding process^16^. As macro-moths are an indicator group for other terrestrial invertebrates^10^, our main findings are likely to be relevant for other invertebrate taxa too. Moreover, moths are key to ecosystem functioning. For instance, the herbivore caterpillars are important nutrient cyclers, flying adults are nocturnal pollinators, and both larvae and adults are important food resources for several other trophic levels^10^.

Summarised, we here show that landscape-scale farmland abandonment can lead to multi-habitat landscapes characterised by high levels of macro-moth diversity, which may translate in a better functioning and more resilient ecosystem than the replaced agricultural system.

## Methods

### Study area

**Figure 4:**
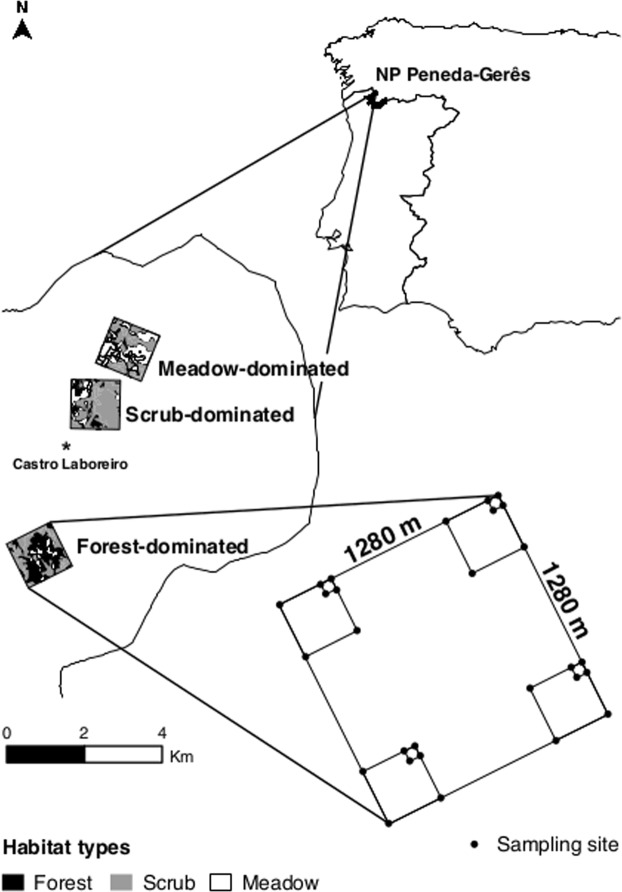
The study area and location of sampling sites in Peneda-Gerês National Park, NW-Portugal. 84 fixed sampling sites were part of a semi-nested sampling design in three study landscapes that represented a farmland abandonment gradient.

The study area was located nearby the town of Castro Laboreiro (42.031 N, −8.155 W) within the National Park of Peneda-Gerês, NW-Portugal (Fig. 4), which is a mountainous region situated in the transitional zone between the Atlantic and Mediterranean ecoregions. The study area (49.7 km2; 750–1155 MASL) consisted of scrub (78.4%), forest (10.5%), agricultural land (9.8%) and urban land (1.3%), with land-use covers obtained by manually digitising aerial photographs (minimum mapping unit: ca. 1000 m^2^)^33–35^. Because of the pronounced combination of physical constraints of the landscape, low agricultural productivity and socio-economic drivers, this remote mountain area has undergone a rural exodus since the 1950’s, which has increased the turnover of agricultural fields to scrub and forest^34,35^.

### Sampling design

Field work was performed across three landscapes of each 1.64 km^2^ representing ecological succession stages after farmland abandonment: meadow-, scrub- and forest-dominated landscapes. For each landscape, 28 fixed circular sampling sites of 300 m^2^ (radius ca. 10 m) were set up using a semi-nested design^36^ (Fig. 4). Each landscape varied in the relative cover of the three habitat types (meadow-dominated landscape: 13 meadow, 13 scrub and 2 forest sites; scrub-dominated landscape: 4 meadow, 20 scrub and 4 forest sites; forest-dominated landscape: 8 scrub and 20 forest sites). As we were not able to confirm the land use history of every single site, these sampling sites represent a spatial succession series which approximates a temporal series since ecological succession within these landscapes turns abandoned meadows into scrub and then into forest. We sampled a total of 17 meadow, 41 scrub, and 26 forest sites. Each site was sampled six times during the main flight season of moths (i.e. May–September): three times in 2011 and another three times in 2012. For analyses, samples of these six sessions were lumped.

Macro-moths were sampled using light traps, which were activated from dusk until dawn. All macro-moth individuals inside and on the trap were enumerated and identified to species-level, with collection for later identification of specimens that could not be identified immediately. One observer (TM) simultaneously sampled seven sites per sampling night, so that they were sampled under identical weather conditions, covering three spatial scales: (i) 20 × 20 m, (ii) 80 × 80 m, and (iii) 320 × 320 m (Fig. 4). Moreover, during each of the six sampling rounds all 84 sites (i.e. twelve sampling nights) were sampled in as short a period as possible so as to avoid seasonal differences in species composition. Sampling was neither conducted during full moon nor during nights close to it, and was only conducted during sufficiently favourable weather conditions for moth flight activity^20,37^. Although the degree by which macro-moths are attracted to light is known to differ among families, the used light-trap type (Heath pattern 6 W actinic; Heath^38^) has an effective attraction radius of typically 10 m, with only very low percentages of moths drawn in from further away^39^. This attraction radius hence translates in a sampling area of ca. 300 m^2^. The possible bias, due to intrinsic differences in flight-to-light behaviour among individuals, species and families, is identical for each of the 84 sites, as they were all sampled with identical light-traps. As such, although local absolute light-trap samples are biased with respect to the local community, the observed relative differences among trap sites were not biased.

Species were grouped as either forest or non-forest species according to whether they displayed a higher relative abundance in forest vs. non-forest (i.e. meadow/scrub) habitat, respectively, while correcting for the different number of forest vs. non-forest sites (i.e. 26 vs. 58 sites, respectively). Species with five or fewer individuals were classified based on literature and expert knowledge. As such, 196 species were classified as forest species and 182 as non-forest species (Supplementary Table S1).

### Gamma and alpha diversities

Effective species numbers were used to characterise the taxonomic diversity for each habitat type at regional and local scales. Effective species numbers are all based on the same generalized entropy formula, differing only by an exponent *q* that determines the sensitivity to species relative abundances^40^, with low values more sensitive to rare species, and high values more sensitive to abundant species. We used the following effective species numbers: species richness (*q* = 0), the exponential of Shannon diversity (*q* = 1), the reciprocal of Simpson’s diversity (*q* = 2) and the reciprocal of the Berger-Parker index (*q* $\to \,\infty$, i.e. reciprocal of the proportional abundance of the commonest species). At sampling site level, we tested for effects of habitat type on each of these metrics and on Simpson’s evenness, by using a bootstrap procedure of 4000 runs and comparing confidence intervals (95%) around the bootstrapped mean of metrics. Simpson’s evenness was included as it is mathematically independent from the reciprocal of Simpson’s diversity^41^.

### Beta diversity

We looked at two aspects of beta diversity: (i) the mean dissimilarity of species composition between any pair of sites for each habitat type, and (ii) how that dissimilarity changes with distance between sites. For the former, we calculated mean Jaccard dissimilarities (hereafter ‘observed beta diversity’; see Supplementary Fig. S2 for mean Sørensen dissimilarities). For the latter, the spatial turnover rate was calculated for each habitat type as the slope of a linear least-squares regression of dissimilarity on geographic distance^42^. We used a bootstrap procedure with 4000 runs to test whether observed beta diversity and turnover rate differed between the three habitat types.

### Corrected beta diversity

As the different habitat types had different values of both mean alpha and gamma diversity, we used a null model to remove the effect of alpha diversity on beta diversity^43^. For each habitat type, we randomised species in the observed data using a null model with 2000 iterations that shuffled species between sampling sites while maintaining the species total fixed (e.g., SIM2 in Gotelli^44^). We then calculated the expected beta diversity between all sampling sites from the same habitat type using the Jaccard index (see Supplementary Figs S2 and S3 for output based on the Sørensen index). Next, beta deviations were calculated for each habitat type as the difference between observed and mean expected beta diversity, divided by the standard deviation of the expected beta diversity^43^. A bootstrap procedure with 4000 iterations was used to test whether the expected beta diversity and beta deviation differed between the three habitat types. The beta deviation represents the beta diversity not accounted for in the random sampling of the meta-community. In other words, the beta deviation represents a standard effect size with positive and negative values indicating more and less beta diversity, respectively, than expected by chance. All analyses were performed using R version 3.1.1 package ‘vegan’^45,46^.

## Supplementary information

Supplementary Information

## Data Availability

The analysed datasets are available from the corresponding author on reasonable request.

## References

[1] Pereira HM, Navarro LM, Martins IS (2012). Global biodiversity change: the bad, the good, and the unknown. Annu. Rev. Environ. Resour..

[2] Karp DS (2012). Intensive agriculture erodes β-diversity at large scales. Ecol. Lett..

[3] Newbold T (2015). Global effects of land use on local terrestrial biodiversity. Nature.

[4] Clewell, A. F. & Aronson, J. *Ecological Restoration: Principles*, *Values*, *and Structure of an Emerging Profession*. (Island Press 2013).

[5] Benayas JR, Martins A, Nicolau JM, Schulz JJ (2007). Abandonment of agricultural land: an overview of drivers and consequences. CAB Rev. Perspect. Agric. Vet. Sci. Nutr. Nat. Resour..

[6] van Swaay, C. *et al*. *European Red List of Butterflies*. (IUCN Regional Office for Europe, 2010).

[7] Fischer J, Hartel T, Kuemmerle T (2012). Conservation policy in traditional farming landscapes. Conserv. Lett..

[8] Bowen ME, McAlpine CA, House APN, Smith GC (2007). Regrowth forests on abandoned agricultural land: a review of their habitat values for recovering forest fauna. Biol. Conserv..

[9] Navarro LM, Pereira HM (2012). Rewilding abandoned landscapes inEurope. Ecosystems.

[10] Merckx, T., Huertas, B., Basset, Y. & Thomas, J. A global perspective on conserving butterflies and moths and their habitats. In *Key Topics in Conservation Biology* 2 (eds Macdonald, D. W. & Willis, K. J.) 237–257 (John Wiley & Sons 2013).

[11] Whittaker RH (1960). Vegetation of the Siskiyou Mountains, Oregon and California. Ecol. Monogr..

[12] Margules CR, Pressey RL (2000). Systematic conservation planning. Nature.

[13] Merckx T (2012). Conserving threatened Lepidoptera: towards an effective woodland management policy in landscapes under intense human land-use. Biol. Conserv..

[14] Boesing AL, Nichols E, Metzger JP (2018). Land use type, forest cover and forest edges modulate avian cross‐habitat spillover. J. Appl. Ecol..

[15] Pöyry J, Lindgren S, Salminen J, Kuussaari M (2004). Restoration of butterfly and moth communities in semi‐natural grasslands by cattle grazing. Ecol. Appl..

[16] Merckx T, Pereira HM (2015). Reshaping agri-environmental subsidies: from marginal farming to large-scale rewilding. Basic Appl. Ecol..

[17] Navarro, L. M., Proença, V., Kaplan, J. O. & Pereira, H. M. Maintaining disturbance-dependent habitats. in *Rewilding European Landscapes* (eds Pereira, H. M. & Navarro, L. M.) 143–167 (Springer International Publishing 2015).

[18] Beck J, Schulze CH, Linsenmair KE, Fiedler K (2002). From forest to farmland: diversity of geometrid moths along two habitat gradients on Borneo. J. Trop. Ecol..

[19] Kivinen S, Luoto M, Kuussaari M, Helenius J (2006). Multi-species richness of boreal agricultural landscapes: effects of climate, biotope, soil and geographical location. J. Biogeogr..

[20] Merckx T, Marini L, Feber RE, Macdonald DW (2012). Hedgerow trees and extended-width field margins enhance macro-moth diversity: implications for management. J. Appl. Ecol..

[21] Warren, M. S. & Key, R. S. Woodlands: past, present and potential for insects. In *The Conservation of Insects and their Habitats* (eds Collins, N. M. & Thomas, J. A.) 155–211 (Academic Press, 1991).

[22] Wagner DL, Nelson MW, Schweitzer DF (2003). Shrubland Lepidoptera of southern New England and southeastern New York: ecology, conservation, and management. For. Ecol. Manag..

[23] Merckx T, Van Dongen S, Matthysen E, Van Dyck H (2008). Thermal flight budget of a woodland butterfly in woodland versus agricultural landscapes: An experimental assessment. Basic Appl. Ecol..

[24] Merckx T (2010). Shelter benefits less mobile moth species: the field-scale effect of hedgerow trees. Agric. Ecosyst. Environ..

[25] Toivonen M (2017). High cover of forest increases the abundance of most grassland butterflies in boreal farmland. Insect Conserv. Divers..

[26] Summerville KS, Boulware MJ, Veech JA, Crist TO (2003). Spatial variation in species diversity and composition of forest Lepidoptera in Eastern Deciduous Forests of North America. Conserv. Biol..

[27] Kessler M (2009). Alpha and beta diversity of plants and animals along a tropical land-use gradient. Ecol. Appl..

[28] Grime JP (1998). Benefits of plant diversity to ecosystems: immediate, filter and founder effects. J. Ecol..

[29] Gaston KJ, Fuller RA (2008). Commonness, population depletion and conservation biology. Trends Ecol. Evol..

[30] Chase JM (2014). Spatial scale resolves the niche versus neutral theory debate. J. Veg. Sci..

[31] Tucker CM, Shoemaker LG, Davies KF, Nemergut DR (2016). & Melbourne, B. ADifferentiating between niche and neutral assembly in metacommunities using null models of β-diversity. Oikos.

[32] Pöyry J, Lindgren S, Salminen J, Kuussaari M (2005). Responses of butterfly and moth species to restored cattle grazing in semi-natural grasslands. Biol. Conserv..

[33] IGP. *Carta De Uso E Ocupação Do Solo De Portugal Continental*. (COS, 2007).

[34] Rodrigues, P. Landscape Changes in Castro Laboreiro: from Farmland Abandonment to Forest Regeneration. (Faculdade de Ciencias da Universidade de Lisboa, 2010).

[35] Beilin R (2014). Analysing how drivers of agricultural land abandonment affect biodiversity and cultural landscapes using case studies from Scandinavia, Iberia and Oceania. Land Use Policy.

[36] Proença V, Pereira HM (2013). Species-area models to assess biodiversity change in multi-habitat landscapes: the importance of species habitat affinity. Basic Appl. Ecol..

[37] Yela JL, Holyoak M (1997). Effects of moonlight and meteorological factors on light and bait trap catches of Noctuid moths (Lepidoptera: Noctuidae). Environ. Entomol..

[38] Heath J (1965). A genuinely portable MV light trap. Entomol Rec J Var.

[39] Merckx T, Slade EM (2014). Macro-moth families differ in their attraction to light: implications for light-trap monitoring programmes. Insect Conserv. Divers..

[40] Hill MO (1973). Diversity and evenness: a unifying notation and its consequences. Ecology.

[41] Smith B, Wilson JB (1996). A consumer’s guide to evenness indices. Oikos.

[42] Anderson MJ (2011). Navigating the multiple meanings of β diversity: a roadmap for the practicing ecologist. Ecol. Lett..

[43] Kraft NJB (2011). Disentangling the drivers of β diversity along latitudinal and elevational gradients. Science.

[44] Gotelli NJ (2000). Null model analysis of species co-occurrence patterns. Ecology.

[45] R Core Team. *R: A Language and Environment for Statistical Computing*. (R Foundation for Statistical Computing, 2014).

[46] Oksanen, J. *et al*. *vegan: Community Ecology Package* (2015).

