# Supplementary material for: Beta diversity patterns reveal positive effects of farmland abandonment on moth communities
Source: Sci Rep. 2019 Feb 7;9:1549. doi: 10.1038/s41598-018-38200-3 (PMC6367322; doi:10.1038/s41598-018-38200-3)

Supplementary Information

Beta diversity patterns reveal positive effects of farmland abandonment on moth communities

Murilo Dantas de Miranda[1,2], Henrique M. Pereira[1,2,3], Martin F.V. Corley[4], Thomas Merckx[5]

[1]Institute of Biology, Martin Luther University Halle Wittenberg, Halle (Saale), Germany

[2]German Centre for Integrative Biodiversity Research (iDiv) Halle-Jena-Leipzig, Leipzig, Germany

[3]Infraestruturas de Portugal Biodiversity Chair, CIBIO/InBIO - Research Network in Biodiversity and Genetic Resources, Instituto Superior de Agronomia, Universidade de Lisboa, Tapada da Ajuda, Lisboa

[4]CIBIO - Research Center in Biodiversity and Genetic Resources, Universidade do Porto, Campus Agrário de Vairão, Vairão, Portugal

[5]Behavioural Ecology and Conservation Group, Biodiversity Research Centre, Earth and Life Institute, Université catholique de Louvain (UCL), Louvain-la-Neuve, Belgium

* Corresponding author: thomas.merckx@uclouvain.be

Supplementary Table S1. Macro-moth species list. The list indicates species group (SG) classification (N: non-forest species; F: forest species) and the raw number –unweighted in line with numbers of sampling sites per habitat type– of observed individuals, both in the three different habitat types (Forest; Scrub; Meadow) as well as overall (Total). Individuals of cryptic species were aggregated into three species-groups (*Cilix AGG*: *C. glaucata*, *C. hispanica* and *C. algirica*; *Stilbia AGG*: *S. anomala* and *S. andalusiaca*; *Watsonalla AGG*: *W. uncinula* and *W. binaria*).

Species	SG	Forest	Scrub	Meadow	Total
Abraxas grossulariata	N	1	11	3	15
Acronicta aceris	F	3	3	1	7
Acronicta auricoma	N	4	10	2	16
Acronicta euphorbiae	N	0	1	0	1
Acronicta leporina	F	0	1	0	1
Acronicta psi	F	0	1	0	1
Acronicta rumicis	F	20	14	3	37
Adactylotis gesticularia	F	40	1	0	41
Aethalura punctulata	F	0	1	0	1
Agriopis marginaria	F	0	1	0	1
Agrotis bigramma	F	21	14	5	40
Agrotis chretieni	N	2	26	38	66
Agrotis exclamationis	N	3	24	69	96
Agrotis ipsilon	N	3	13	4	20
Agrotis puta	N	1	2	0	3
Agrotis segetum	F	46	59	13	118
Agrotis trux	N	115	200	78	393
Alcis repandata	F	115	104	11	230
Aleucis distinctata	N	0	1	1	2
Amphipyra pyramidea	F	18	5	1	24
Anarta myrtilli	N	2	21	1	24
Antitype chi	N	0	10	2	12
Apamea lithoxylaea	N	1	3	6	10
Apamea monoglypha	N	29	72	99	200
Apamea remissa	N	2	11	3	16
Apamea scolopacina	N	0	1	1	2
Aplocera efformata	F	4	4	0	8
Aplocera plagiata	N	3	2	0	5
Aporophyla nigra	N	2	3	3	8

Species	SG	Forest	Scrub	Meadow	Total
Arctia caja	N	2	4	5	11
Aspitates gilvaria	N	0	0	3	3
Atlantarctia tigrina	F	34	33	0	67
Autographa gamma	N	1	18	5	24
Autophila cataphanes	N	1	1	0	2
Biston betularia	N	4	9	4	17
Biston strataria	F	0	1	0	1
Brachylomia viminalis	N	3	3	4	10
Bryophila domestica	N	1	2	1	4
Bryophila ravula	F	18	9	8	35
Cabera exanthemata	F	5	3	0	8
Cabera pusaria	F	10	3	1	14
Calamia tridens	N	0	0	4	4
Calliteara pudibunda	N	3	6	1	10
Callopistria juventina	F	1	0	0	1
Callopistria latreillei	F	1	0	0	1
Calophasia hamifera	F	1	1	0	2
Calophasia platyptera	N	1	0	0	1
Campaea margaritaria	F	40	9	2	51
Camptogramma bilineata	F	14	3	9	26
Caradrina selini	F	256	147	55	458
Caradrina aspersa	F	86	15	1	102
Caradrina clavipalpis	N	1	1	0	2
Caradrina flavirena	N	1	3	0	4
Caradrina morpheus	N	2	34	19	55
Catarhoe rubidata	N	0	0	4	4
Catephia alchymista	F	1	0	0	1
Catocala nupta	F	2	3	1	6
Catocala optata	N	1	3	1	5
Catocala promissa	F	36	4	0	40
Catocala sponsa	F	5	2	0	7
Cerastis rubricosa	N	5	32	11	48
Cerura iberica	N	0	0	1	1
Charanyca ferruginea	N	121	213	60	394
Charissa avilarius	F	8	0	0	8
Charissa mucidaria	F	27	14	1	42
Charissa obscurata	F	51	33	2	86
Charissa predotae	F	89	32	1	122
Chemerina caliginearia	N	0	1	0	1
Chesias isabella	N	6	14	10	30
Chloantha hyperici	N	0	1	0	1
Chloroclysta siterata	F	1	0	0	1
Chloroclystis v-ata	F	3	1	1	5

Species	SG	Forest	Scrub	Meadow	Total
Cilix AGG	F	5	0	0	5
Cleonymia diffluens	F	28	21	2	51
Cleora cinctaria	N	1	2	1	4
Cleorodes lichenaria	F	6	1	1	8
Clostera pigra	F	0	1	0	1
Colocasia coryli	F	4	3	2	9
Colostygia hilariata	N	0	1	1	2
Colostygia pectinataria	N	0	2	2	4
Comibaena bajularia	F	1	0	0	1
Compsoptera opacaria	F	8	4	0	12
Conisania andalusica	F	12	1	1	14
Conistra rubiginea	F	4	4	3	11
Coscinia cribraria	F	196	131	40	367
Cosmia trapezina	F	9	5	0	14
Cosmorhoe ocellata	N	12	20	23	55
Crocallis albarracina	F	17	11	0	28
Crocallis dardoinaria	F	36	9	0	45
Crocallis elinguaria	F	9	0	1	10
Crocallis tusciaria	N	1	0	0	1
Cryphia algae	F	15	3	0	18
Cryphia pallida	F	3	0	0	3
Cucullia umbratica	N	0	0	2	2
Cybosia mesomella	N	4	17	4	25
Cyclophora albipunctata	F	3	2	1	6
Cyclophora linearia	F	1	0	0	1
Cyclophora porata	F	24	7	0	31
Cyclophora punctaria	F	38	11	2	51
Cyclophora puppillaria	F	9	8	4	21
Cyclophora ruficiliaria	F	91	11	1	103
Cymatophorina diluta	F	1	2	0	3
Cymbalophora pudica	F	14	14	2	30
Deilephila porcellus	N	0	2	4	6
Denticucullus pygmina	F	18	2	1	21
Diachrysia chrysitis	N	1	1	0	2
Diacrisia sannio	N	3	57	12	72
Diaphora mendica	F	27	10	7	44
Diarsia brunnea	F	3	2	0	5
Diarsia guadarramensis	N	4	10	3	17
Diarsia rubi	N	1	0	0	1
Drepana curvatula	F	0	2	2	4
Drymonia querna	N	1	4	2	7
Drymonia ruficornis	F	4	4	2	10
Drymonia velitaris	F	5	0	1	6

Species	SG	Forest	Scrub	Meadow	Total
Dryobotodes roboris	N	2	7	0	9
Dypterygia scabriuscula	F	4	2	4	10
Dyscia distinctaria	N	0	5	3	8
Dysgonia algira	F	9	0	0	9
Dyspessa ulula	N	2	2	0	4
Dysstroma citrata	F	5	4	0	9
Dysstroma truncata	F	2	3	1	6
Ectropis crepuscularia	F	3	4	0	7
Egira conspicillaris	F	6	2	0	8
Eilema caniola	F	59	30	12	101
Eilema complana	N	84	115	174	373
Eilema lurideola	N	20	32	22	74
Eilema pygmaeola	N	1	12	5	18
Eilema uniola	F	455	56	7	518
Elaphria venustula	N	0	0	1	1
Electrophaes corylata	F	1	1	0	2
Ematurga atomaria	N	1	4	0	5
Ennomos alniaria	F	4	1	3	8
Ennomos erosaria	F	1	0	0	1
Entephria cyanata	N	2	0	0	2
Epilecta linogrisea	F	19	0	0	19
Epirrhoe alternata	N	10	16	14	40
Epirrhoe galiata	F	14	17	4	35
Episema glaucina	N	0	2	0	2
Eublemma ostrina	N	0	1	0	1
Eublemma polygramma	N	0	0	1	1
Eublemma purpurina	N	1	1	0	2
Eugnorisma arenoflavida	F	21	4	1	26
Eugnorisma glareosa	N	208	355	120	683
Euphyia frustata	N	2	0	1	3
Eupithecia abbreviata	F	2	1	0	3
Eupithecia centaureata	N	1	1	1	3
Eupithecia dodoneata	F	0	1	0	1
Eupithecia extraversaria	F	3	0	0	3
Eupithecia icterata	F	6	0	7	13
Eupithecia innotata	N	0	1	0	1
Eupithecia laquaearia	F	12	4	1	17
Eupithecia nanata	N	14	33	1	48
Eupithecia pantellata	F	1	0	0	1
Eupithecia pulchellata	F	16	14	10	40
Eupithecia pyreneata	F	8	0	0	8
Eupithecia scopariata	F	287	260	34	581
Eupithecia subfuscata	N	10	22	8	40

Species	SG	Forest	Scrub	Meadow	Total
Eupithecia venosata	F	4	2	1	7
Eupithecia vulgata	F	17	18	7	42
Euplagia quadripunctaria	F	44	12	1	57
Euplexia lucipara	N	1	4	3	8
Euproctis chrysorrhoea	F	155	6	3	164
Euproctis similis	F	0	1	1	2
Euxoa obelisca	F	18	19	8	45
Euxoa tritici	N	10	20	3	33
Falcaria lacertinaria	F	3	1	4	8
Geometra papilionaria	F	1	0	0	1
Gnophos furvata	N	4	0	0	4
Gnophos obfuscata	N	0	0	1	1
Griposia aprilina	F	2	0	0	2
Gymnoscelis rufifasciata	F	257	188	215	660
Habrosyne pyritoides	N	2	2	5	9
Hadena albimacula	N	1	0	1	2
Hadena bicruris	N	2	2	1	5
Hadena confusa	N	0	1	0	1
Hadena filograna	N	3	7	0	10
Hadena perplexa	F	7	1	4	12
Harpyia milhauseri	F	5	6	0	11
Helicoverpa armigera	N	0	2	0	2
Heliothis nubigera	N	1	0	0	1
Heliothis peltigera	N	0	1	1	2
Heterogynis paradoxa	N	0	1	0	1
Hoplodrina ambigua	F	52	73	15	140
Hoplodrina hesperica	F	333	111	52	496
Hoplodrina octogenaria	N	105	286	196	587
Hydriomena furcata	F	48	3	7	58
Hydriomena impluviata	F	4	2	0	6
Hyles livornica	N	0	1	2	3
Hyphoraia dejeani	N	26	41	40	107
Hypomecis punctinalis	F	4	0	0	4
Hypomecis roboraria	F	1	0	0	1
Idaea alyssumata	F	7	0	0	7
Idaea aversata	F	30	7	1	38
Idaea belemiata	N	1	0	1	2
Idaea biselata	N	1	2	2	5
Idaea calunetaria	F	4	2	0	6
Idaea cervantaria	N	0	0	1	1
Idaea contiguaria	F	151	81	47	279
Idaea degeneraria	F	68	40	2	110
Idaea deversaria	F	33	1	0	34

Species	SG	Forest	Scrub	Meadow	Total
Idaea dromikos	F	1	0	0	1
Idaea eugeniata	F	33	9	0	42
Idaea fuscovenosa	F	14	4	3	21
Idaea humiliata	N	0	2	0	2
Idaea infirmaria	N	1	0	0	1
Idaea joannisiata	N	2	2	0	4
Idaea litigiosaria	N	1	2	0	3
Idaea lutulentaria	F	7	1	0	8
Idaea macilentaria	N	1	4	23	28
Idaea moniliata	N	1	0	0	1
Idaea obsoletaria	F	12	1	0	13
Idaea ostrinaria	F	4	3	0	7
Idaea rubraria	N	2	0	0	2
Idaea sardoniata	N	1	3	0	4
Idaea straminata	F	15	2	0	17
Idaea subsaturata	N	1	1	0	2
Idaea subsericeata	F	12	15	10	37
Isturgia miniosaria	N	22	173	50	245
Jodis lactearia	F	5	1	0	6
Lacanobia contigua	N	1	10	5	16
Lacanobia oleracea	N	1	3	1	5
Lacanobia thalassina	N	11	21	6	38
Lacanobia w-latinum	N	6	13	1	20
Laothoe populi	N	0	5	1	6
Lasiocampa quercus	N	2	7	8	17
Lasiocampa trifolii	N	2	14	0	16
Leucania loreyi	N	0	2	0	2
Leucania putrescens	F	29	20	6	55
Leucochlaena oditis	N	5	5	14	24
Leucoma salicis	N	0	1	0	1
Litoligia literosa	N	1	2	0	3
Lomaspilis marginata	F	13	4	2	19
Lophoterges millierei	F	9	3	0	12
Luperina nickerlii	N	2	5	4	11
Luperina testacea	N	71	139	280	490
Lycophotia erythrina	F	326	342	73	741
Lycophotia molothina	F	54	73	3	130
Lycophotia porphyria	N	23	159	25	207
Lymantria dispar	F	4	1	0	5
Lymantria monacha	F	18	0	1	19
Macaria alternata	F	33	26	2	61
Macaria notata	F	1	0	0	1
Macrothylacia rubi	N	4	7	4	15

Species	SG	Forest	Scrub	Meadow	Total
Malacosoma castrensis	N	0	0	1	1
Malacosoma neustria	N	1	4	1	6
Mamestra brassicae	N	0	1	0	1
Meganola strigula	F	19	3	1	23
Melanchra persicariae	N	1	1	0	2
Melanchra pisi	N	0	4	6	10
Menophra abruptaria	F	23	13	9	45
Menophra nycthemeraria	F	4	2	0	6
Mesapamea secalis	N	4	5	8	17
Mesoligia furuncula	N	3	3	8	14
Mesotype didymata	N	0	7	5	12
Miltochrista miniata	F	1	0	0	1
Mimas tiliae	F	1	0	0	1
Minucia lunaris	F	1	0	0	1
Mormo maura	F	4	0	0	4
Mythimna albipuncta	F	13	16	5	34
Mythimna conigera	N	8	67	87	162
Mythimna ferrago	F	59	67	59	185
Mythimna impura	N	25	148	65	238
Mythimna l-album	N	2	0	2	4
Mythimna sicula	F	15	17	15	47
Mythimna unipuncta	N	2	0	0	2
Mythimna vitellina	N	55	156	30	241
Noctua comes	N	12	33	13	58
Noctua interjecta	N	4	17	16	37
Noctua janthe	F	7	8	4	19
Noctua orbona	F	6	3	0	9
Noctua pronuba	N	94	304	44	442
Noctua tirrenica	F	14	13	1	28
Notodonta dromedarius	F	0	0	1	1
Nychiodes andalusiaria	F	46	13	5	64
Nycteola revayana	F	2	1	0	3
Nycteola siculana	F	1	0	0	1
Nyctobrya muralis	F	9	0	0	9
Ochropleura leucogaster	N	0	3	4	7
Ochropleura plecta	F	5	5	6	16
Ocneria rubea	F	12	3	0	15
Odice pergrata	N	1	3	0	4
Oligia strigilis	N	39	47	64	150
Oligia versicolor	N	1	0	0	1
Olivenebula xanthochloris	F	20	2	0	22
Omphaloscelis lunosa	N	38	64	569	671
Opisthograptis luteolata	F	2	0	0	2

Species	SG	Forest	Scrub	Meadow	Total
Orthosia cerasi	N	5	9	3	17
Orthosia gothica	N	21	95	44	160
Orthosia incerta	N	9	18	7	34
Orthosia miniosa	N	2	6	2	10
Ourapteryx sambucaria	F	2	0	0	2
Pachycnemia hippocastanaria	F	661	753	161	1575
Pachycnemia tibiaria	F	131	151	14	296
Paidia rica	F	2	1	0	3
Panolis flammea	F	0	1	0	1
Paracolax tristalis	F	5	0	0	5
Pasiphila rectangulata	F	1	0	0	1
Perconia baeticaria	N	22	76	30	128
Peribatodes ilicaria	F	37	12	7	56
Peribatodes rhomboidaria	F	51	21	38	110
Peridea anceps	F	11	4	3	18
Peridroma saucia	F	5	8	1	14
Perizoma hydrata	F	3	3	0	6
Petrophora chlorosata	N	34	68	8	110
Phalera bucephala	F	4	0	1	5
Pharmacis lupulina	F	5	1	0	6
Phlogophora meticulosa	N	0	5	1	6
Photedes minima	N	0	2	2	4
Phragmatobia fuliginosa	N	4	24	14	42
Plagodis dolabraria	F	0	1	0	1
Polia nebulosa	F	6	2	0	8
Polymixis argillaceago	N	1	3	0	4
Polymixis dubia	F	23	12	0	35
Polymixis flavicincta	N	0	0	1	1
Polymixis lichenea	N	2	8	7	17
Polymixis xanthomista	N	3	30	7	40
Polyploca ridens	N	2	4	1	7
Pseudenargia ulicis	F	14	12	0	26
Pseudoips prasinana	F	3	0	1	4
Pseudoterpna coronillaria	F	92	96	41	229
Psilogaster loti	N	0	1	0	1
Rhodometra sacraria	N	0	1	0	1
Rhodostrophia calabra	F	25	9	0	34
Rhodostrophia vibicaria	F	11	10	0	21
Rhoptria asperaria	N	64	197	4	265
Rivula sericealis	N	0	1	1	2
Saturnia pavonia	N	0	2	1	3
Scoliopteryx libatrix	N	0	0	2	2
Scopula asellaria	N	0	3	0	3

Species	SG	Forest	Scrub	Meadow	Total
Scopula imitaria	F	59	14	0	73
Scopula marginepunctata	N	1	0	0	1
Scopula rufomixtaria	N	1	0	0	1
Scotopteryx coelinaria	N	20	36	14	70
Scotopteryx luridata	N	0	11	3	14
Scotopteryx peribolata	N	13	80	17	110
Selenia dentaria	N	2	11	1	14
Selenia lunularia	F	8	2	1	11
Selidosema pyrenaearia	F	21	22	1	44
Selidosema taeniolaria	F	63	38	12	113
Sesamia nonagrioides	N	2	0	0	2
Sideridis reticulata	N	0	0	1	1
Sideridis rivularis	F	2	4	0	6
Sphinx ligustri	N	1	2	0	3
Spilosoma lutea	F	17	13	6	36
Spudaea ruticilla	N	2	4	4	10
Stauropus fagi	F	1	1	0	2
Stilbia AGG	F	54	84	19	157
Tephronia lhommaria	F	13	2	0	15
Tephronia sepiaria	N	0	0	15	15
Thalera fimbrialis	F	5	1	0	6
Thalpophila vitalba	N	11	65	35	111
Thaumetopoea pityocampa	F	1	1	1	3
Thera obeliscata	N	0	3	0	3
Tholera decimalis	N	4	47	110	161
Thyatira batis	F	2	0	0	2
Timandra comae	N	0	1	1	2
Trichiura castiliana	F	7	5	0	12
Trichoplusia ni	N	1	0	0	1
Trichopteryx carpinata	N	0	0	1	1
Trigonophora crassicornis	N	1	0	0	1
Trigonophora flammea	F	36	30	9	75
Trigonophora haasi	N	25	129	97	251
Trigonophora jodea	F	14	22	2	38
Watsonalla AGG	F	11	5	0	16
Watsonarctia deserta	N	0	3	1	4
Xanthia icteritia	N	1	3	0	4
Xanthia togata	F	2	0	0	2
Xanthorhoe fluctuata	N	0	2	2	4
Xanthorhoe iberica	F	5	4	1	10
Xestia agathina	N	455	1226	197	1878
Xestia baja	F	53	70	21	144
Xestia c-nigrum	F	2	3	1	6

Species	SG	Forest	Scrub	Meadow	Total
Xestia castanea	F	164	62	7	233
Xestia xanthographa	N	108	474	318	900
Xylocampa areola	N	1	1	3	5
Zanclognatha lunalis	F	2	0	0	2
Zanclognatha tarsipennalis	F	1	2	0	3

Supplementary Fig. S1. Species accumulation curves for the three habitat types: forest, scrub and meadow.

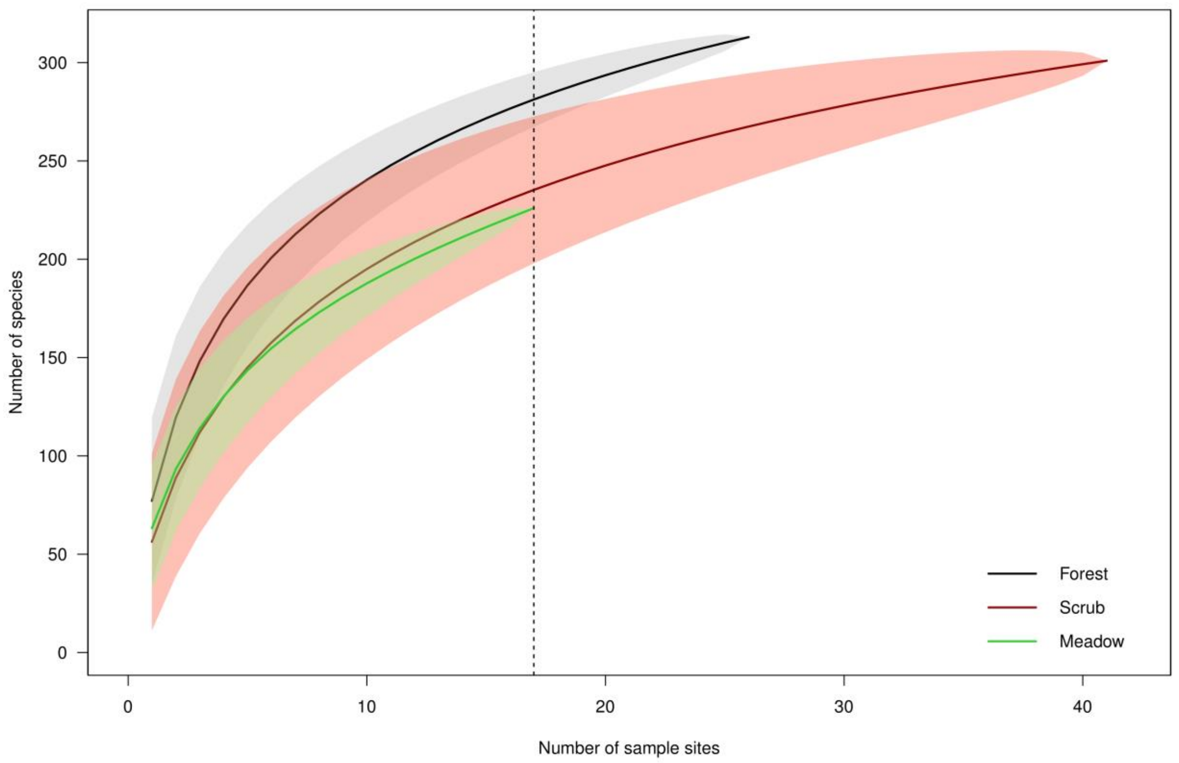

Supplementary Fig. S2. Beta diversity across habitats and species groups. (**A**) Observed beta diversity (*in casu* Sørensen index), (**B**) expected beta diversity from a null model based on random sampling from the regional species pool, and (**C**) beta deviations (standardized effect sizes which represent the difference between observed beta diversity and expected beta diversity) for all species (left), forest species (centre) and non-forest species (right). Beta deviations are positive, indicating beta diversity higher than expected by chance. Dark-, medium- and light-grey bars represent forest, scrub and meadow habitats, respectively. Error bars are 95% confidence intervals based on 4000 bootstrap replicates. Different letters represent statistic differences.

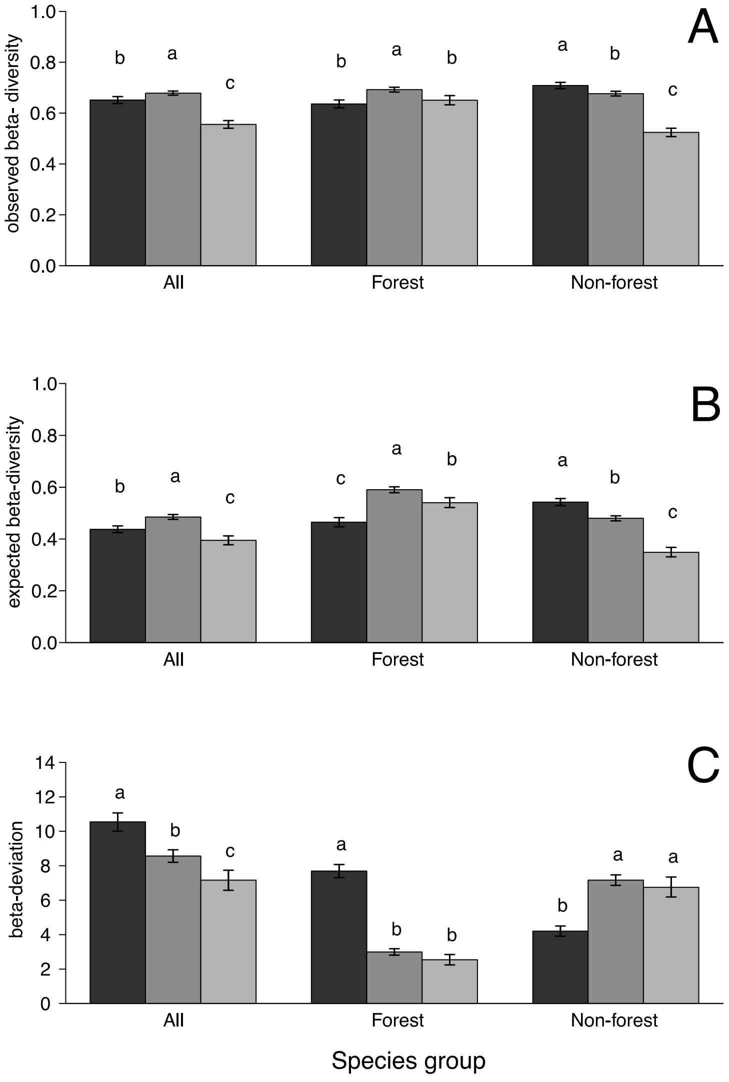

Supplementary Fig. S3. Relationships between geographic distance and beta diversity (*in casu* Sørensen index) for pairs of sampling sites across habitats and species groups. (**A**) Observed beta diversity for all species, (**B**) forest species, and (**C**) non-forest species. Dark-, medium- and light-grey dots represent forest, scrub and meadow habitats, respectively. The solid lines represent the best-fit lines from linear regression. The turnover rates (slope), R^2-values and significance levels of turnover rates (*** $P < 0.001$, ** $P < 0.01$ and * $P < 0.05$) are given within each panel.

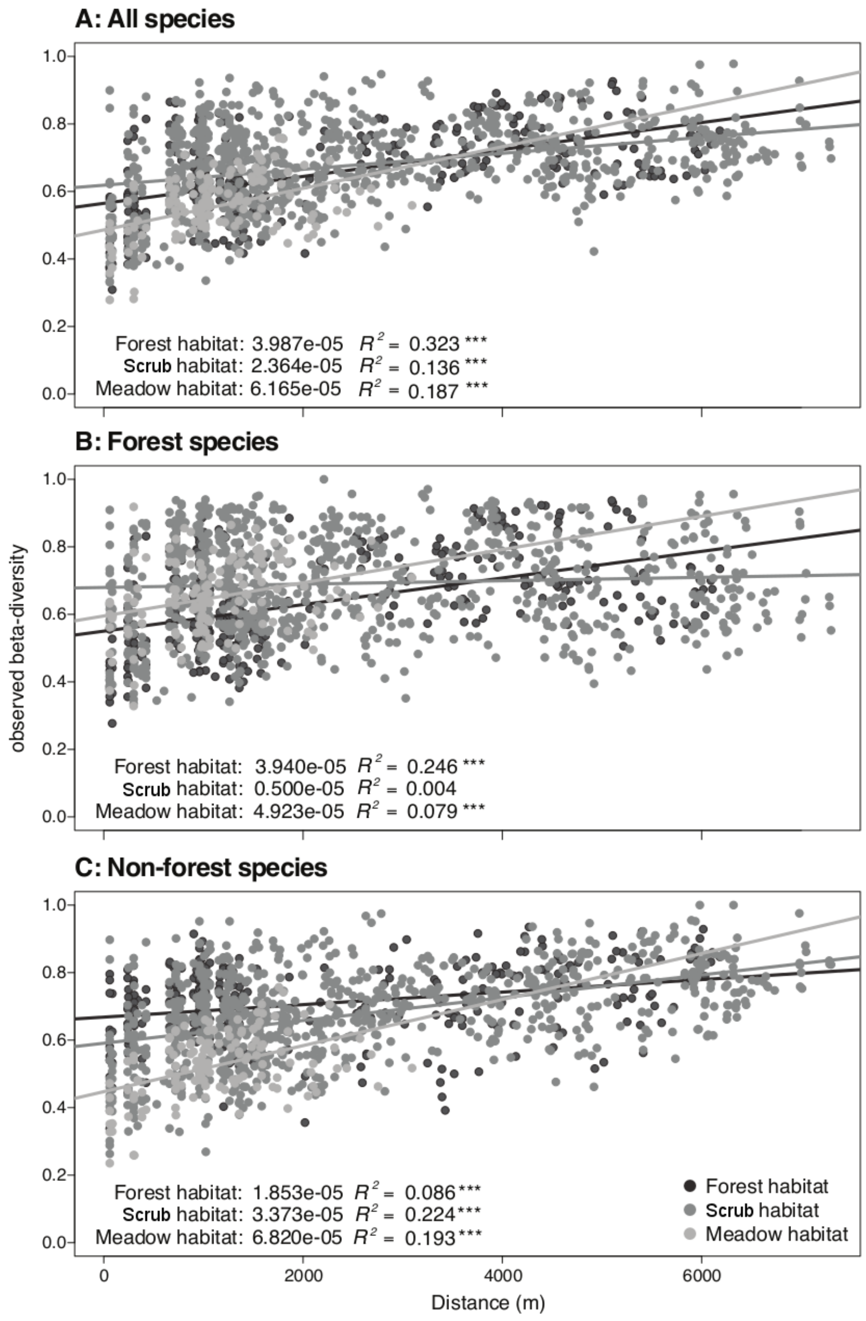

Supplement: Supplementary file 1 — Supplementary Information [file 41598_2018_38200_MOESM1_ESM.pdf]
